# Declining Rates of Inpatient Parathyroidectomy for Primary Hyperparathyroidism in the US

**DOI:** 10.1371/journal.pone.0161192

**Published:** 2016-08-16

**Authors:** Sun Moon Kim, Aimee D. Shu, Jin Long, Maria E. Montez-Rath, Mary B. Leonard, Jeffrey A. Norton, Glenn M. Chertow

**Affiliations:** 1 Division of Nephrology, Department of Medicine, Stanford University School of Medicine, Stanford, California, United States of America; 2 Division of Endocrinology, Gerontology and Metabolism, Stanford University School of Medicine, Stanford, California, United States of America; 3 Division of Pediatric Nephrology, Department of Pediatrics, Stanford University School of Medicine, Stanford, California, United States of America; 4 Department of Surgery, Stanford University School of Medicine, Stanford, California, United States of America; Universidade de Sao Paulo, BRAZIL

## Abstract

Parathyroidectomy is the only curative therapy for patients with primary hyperparathyroidism. However, the incidence, correlates and consequences of parathyroidectomy for primary hyperparathyroidism across the entire US population are unknown. We evaluated temporal trends in rates of inpatient parathyroidectomy for primary hyperparathyroidism, and associated in-hospital mortality, length of stay, and costs. We used the Healthcare Cost and Utilization Project Nationwide Inpatient Sample (NIS) from 2002–2011. Parathyroidectomies for primary hyperparathyroidism were identified using International Classification of Diseases, Ninth Revision codes. Unadjusted and age- and sex- adjusted rates of inpatient parathyroidectomy for primary hyperparathyroidism were derived from the NIS and the annual US Census. We estimated 109,583 parathyroidectomies for primary hyperparathyroidism between 2002 and 2011. More than half (55.4%) of patients were younger than age 65, and more than three-quarters (76.8%) were female. The overall rate of inpatient parathyroidectomy was 32.3 cases per million person-years. The adjusted rate decreased from 2004 (48.3 cases/million person-years) to 2007 (31.7 cases/million person-years) and was sustained thereafter. Although inpatient parathyroidectomy rates declined over time across all geographic regions, a steeper decline was observed in the South compared to other regions. Overall in-hospital mortality rates were 0.08%: 0.02% in patients younger than 65 years and 0.14% in patients 65 years and older. Inpatient parathyroidectomy rates for primary hyperparathyroidism have declined in recent years.

## Introduction

Primary hyperparathyroidism (pHPT) is a common endocrine disorder characterized by chronically elevated serum calcium and increased or inappropriately normal parathyroid hormone (PTH) concentrations. With the advent of routine measurements of serum calcium levels in the United States (US) in the early 1970s, the incidence and prevalence of the disease were found to be much higher than previous estimates [1]. In addition, the clinical profile has shifted from a symptomatic disorder, with hypercalcemic symptoms, kidney stones, or overt bone disease, toward an asymptomatic state, which represents the majority nowadays [2,3]. Parathyroidectomy is the only definitive therapy for patients with pHPT. Parathyroidectomy generally cures the disease, decreases the risk of kidney stones, and improves bone mineral density (BMD) [4–6]. Parathyroidectomy may also improve quality of life and psychological function [6,7]. There has been general agreement that parathyroidectomy is indicated for symptomatic patients, while appropriate management of asymptomatic patients has been a matter of debate [8]. Guidelines for management of asymptomatic primary hyperparathyroidism were developed based on risk for end-organ effects and disease progression, and have changed considerably after each of the Consensus Conferences in 1990, 2002, 2008, and 2013 (Table 1) [9–12]. With respect to referral for surgical intervention, the 2002 guidelines suggested two modifications: lowering the threshold serum calcium concentration to 1.0 mg/dL greater than the upper limit of normal and lowering the bone densitometry consideration to a T-score of <−2.5 at any site [10]. The 2008 guidelines offered three more suggestions: previous fragility fracture represents an indication for parathyroidectomy, but hypercalciuria was no longer regarded as an indication; and impaired kidney function was defined by creatinine clearance below 60 mL/min [11]. The 2013 guidelines recommended radiographic assessment of the spine for fracture, radiographic assessment of the kidneys for nephrolithiasis or nephrocalcinosis, and replaced the recommendation for evaluation of a 24-hour urine calcium [12]. In 2004, the calcimimetic cinacalcet was introduced in the US for secondary hyperparathyroidism, and by 2011 gained additional US Food and Drug Administration (FDA) approval for patients with primary hyperparathyroidism complicated by severe hypercalcemia but unable to undergo surgery.

**Table 1:** Guidelines for surgery in asymptomatic pHPT: a comparison of current recommendations with previous ones.

	1990	2002	2008	2013
Measurement				
Serum Calcium (> upper limit of normal)	1–1.6 mg/dL	1.0 mg/dL	1.0 mg/dL	1.0 mg/dL
Skeletal	BMD by DXA: Z-score < -2.0 (site unspecified)	BMD by DXA: T-score < -2.5 at any site	BMD by DXA: T-score < -2.5 at any site	A. BMD by DXA: T-score < -2.5 at lumbar spine, total hip, femoral neck, or distal 1/3 radius
				B. Vertebral fracture by x-ray, CT, MRI, or VFA
Renal	A. eGFR reduced by >30% from expected	A. eGFR reduced by >30% from expected	A. eGFR < 60 cc/min	A. Creatinine clearance < 60 cc/min
	B. 24-h urine for calcium >400 mg/d	B. 24-h urine for calcium >400 mg/d	B. 24-h urine for calcium not recommended	B. 24-h urine for calcium >400 mg/d and increased stone risk by biochemical stone risk analysis
				C. presence of nephrolithiasis or nephrocalcinosis by x-ray, ultrasound, or CT
Age, y	<50	<50	<50	<50

Abbreviations: eGFR, estimated glomerular filtration rate; MRI, magnetic resonance imaging.

Adapted from Bilezikian *et al*. (JCEM 2014 99(10): 3561–3569)Patients need to meet only one of these criteria to be advised to have parathyroid surgery. They do not have to meet more than one. Surgery is also indicated in patients for whom medical surveillance is neither desired nor possible and in patients opting for surgery, in the absence of meeting any guidelines, as long as there are no medical contraindications.

Several recent studies have reported on the epidemiology of pHPT [1,13–17], yet few reported on parathyroidectomy rates [14,15]. Most previously published studies examined parathyroidectomy within a single academic medical center or insurer; trends in parathyroidectomy for pHPT over time and among different patient groups are unknown. Using data from the Healthcare Cost and Utilization Project Nationwide Inpatient Sample (NIS), we sought to determine temporal trends in the incidence, correlates and complications of inpatient parathyroidectomy for pHPT.

## Materials and Methods

### Study Population

We performed this study using 2002–2011 data from the NIS, the largest, publicly available all-payer database of hospital inpatient stays in the US. The NIS is an administrative dataset created by the Agency for Healthcare Research and Quality from data contributed by participating states [18]. The NIS includes all discharge records for the sampled hospitals and data for approximately 8 million inpatient stays from about 1000 hospitals to approximate a 20% stratified sample of all US hospitals, with the exception of Veterans Affairs hospitals, long-term non-acute care hospitals and chemical dependence or alcohol treatment facilities. Numbers of annual discharges were weighted to generate national estimates for each year. The NIS includes information on all patients regardless of payer, including persons covered by Medicare, Medicaid, private insurance, and the uninsured. Each hospitalization is treated as an individual entry in the database and is coded with one principal diagnosis, up to 24 secondary diagnoses, and 15 procedural diagnoses associated with that stay.

### Case Definition

Cases of parathyroidectomy for pHPT were identified using relevant International Classification of Diseases, Ninth Revision, Clinical Modification (ICD-9-CM) codes. We included all admissions with a procedural diagnosis for parathyroidectomy using ICD-9-CM codes of 06.81 and 06.89, indicating parathyroidectomy was performed during the hospitalization. Among all admissions for parathyroidectomy, we excluded cases which had codes indicating thyroid or parathyroid cancer (193 and 194.1, respectively), because parathyroidectomy could be performed in the setting of cancer even if the patient did not have hyperparathyroidism. Considering that surgical treatment would be suggested for patients with end-stage renal disease (ESRD) (and possibly those with advanced chronic kidney disease) who had refractory secondary hyperparathyroidism (sHPT), we also excluded cases if there were relevant diagnostic and procedural codes for renal failure, ESRD, dialysis, kidney transplantation, and renal hyperparathyroidism during the same hospitalization (S1 Table). To avoid contamination of the sample by the inclusion of inadvertent parathyroidectomy during other surgical procedures, we included patients who had both parathyroidectomy procedural codes and parathyroid-related diagnostic codes. Parathyroid-related diagnostic codes were 1) pHPT, multinodular endocrine neoplasia (MEN), or hypercalcemia, 2) nephrolithiasis/nephrocalcinosis, 3) other parathyroid disease, or 4) parathyroid nodule (S1 Table).

#### Study Variables and Study Outcomes

We ascertained basic demographic variables and hospital information, and identified the following comorbidities using ICD-9-CM codes: diabetes mellitus, hypertension, osteoporosis, fracture, depression, and anxiety (S1 Table). We focused our analysis on rates of parathyroidectomy for pHPT over time and trends of clinical characteristics associated with parathyroidectomy. Subgroup analyses included parathyroidectomy rates by age group (0–44, 45–64, 65+ years old), sex, and geographic region (Northeast, Midwest, South, and West). Total hospital charges were converted to costs using cost-to-charge ratio files in NIS database [19]. Costs reflect the actual costs of production, while charges represent what the hospital billed for the case. The cost-to-charge files contain a hospital-wide cost-to-charge ratio for each hospital. We further examined in-hospital mortality, length of stay, and costs.

### Statistical Analyses

We estimated the weighted total number of hospitalizations with parathyroidectomy for pHPT in each calendar year, applying appropriate discharge-level sampling weights to account for the NIS sampling scheme. We summarized baseline characteristics by year (2002–2011). Medians and interquartile ranges were used for continuous variables and percentages for categorical variables. *P*-value for linear trend across years was computed using logistic regression with year (as a continuous variable) as the sole predictor. To calculate population incidence rates, we divided the national estimates of hospitalizations with parathyroidectomy by the estimated US population from the US Census Current Population Survey data [20]. We performed subgroup analyses by age group, sex, and region in a similar fashion. We calculated age group- and sex- adjusted parathyroidectomy rates using the 2002 Census data as the standard population and its age group-sex distribution as standard weights. Standard errors for the adjusted incidence rates were calculated following the methods of Anderson *et al*. [21]. We determined in-hospital mortality rates, lengths of stay, and associated costs among patients who underwent parathyroidectomy for pHPT. All estimates presented account for the complex survey design (weighting and stratification). We created the cohort using the SAS software (SAS Institute, Cary, NC), and conducted the analyses using SAS and StataMP, version 11 (StataCorp, College Station, TX).

## Results

### Population Characteristics

**Fig 1:**
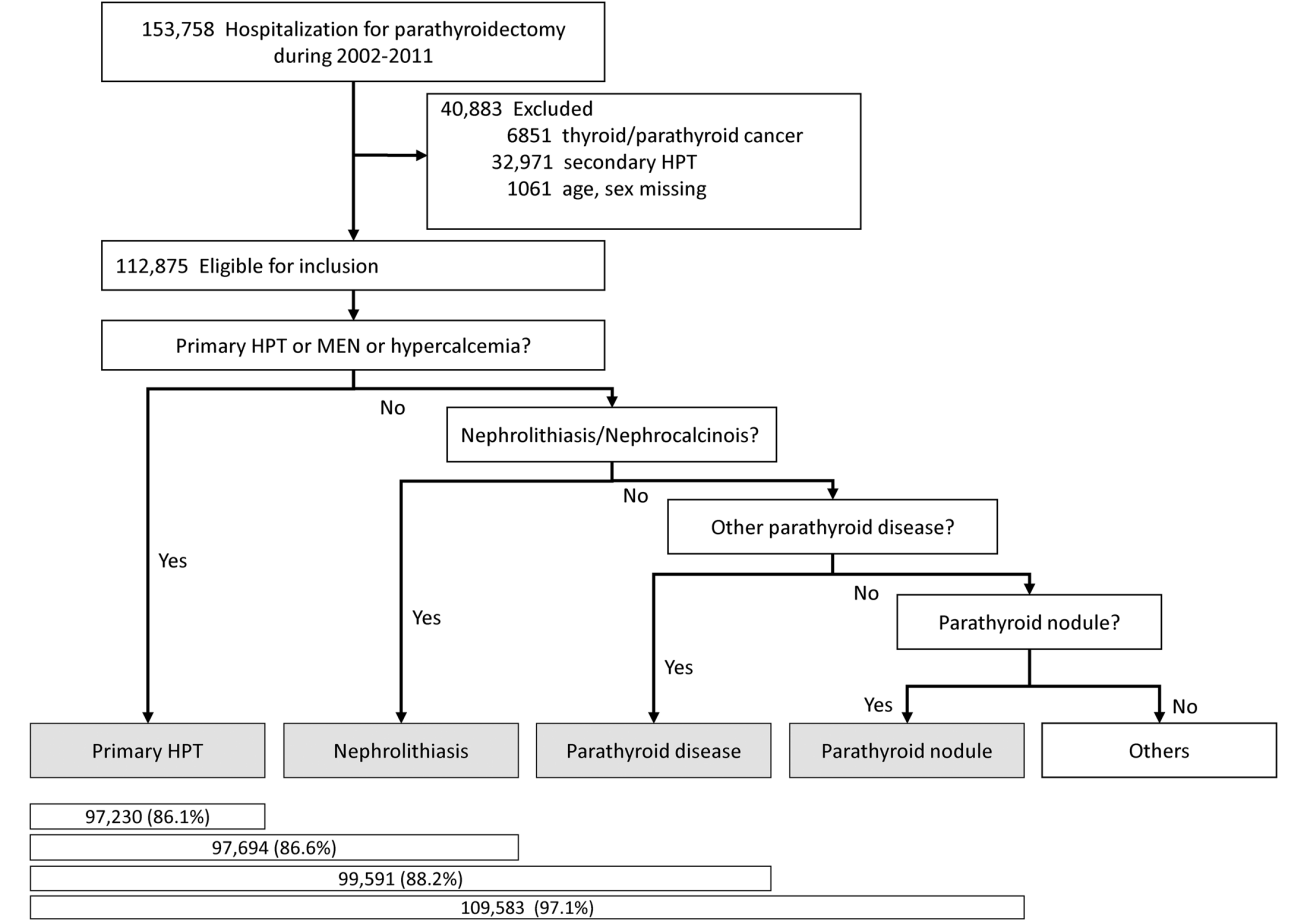
Flow diagram of construction of analytic cohort.

We identified 24,012 hospitalizations including parathyroidectomy, excluding thyroid or parathyroid cancer, sHPT, or age/sex missing from the NIS between 2002 and 2011. More than 97% of eligible patients had an ICD-9-CM code specific for parathyroid disease. Excluding the cases without parathyroid-related diagnostic codes, 23,212 cases remained, which extrapolates to an approximate total of 109,583 parathyroidectomies for pHPT for the entire US population (Fig 1). Table 2 describes baseline characteristics of the study population in each year. More than half (55.4%) of patients undergoing parathyroidectomy for pHPT were younger than age 65; more than three-quarters (76.8%) were female. Mean age, and prevalence of diabetes mellitus and hypertension increased over time. Fewer than one in ten (8.7%) patients underwent complete parathyroidectomy, and the proportion of complete parathyroidectomy did not change significantly over time (*P* for trend = 0.204). The most common ICD-9-CM diagnostic code was hyperparathyroidism (252.0, 82.3%; Table 3), and the second most common code was benign neoplasm of parathyroid gland (227.1, 62.9%). We also evaluated the frequency of pHPT-related comorbidities. Among patients undergoing parathyroidectomy, 0.6% of patients had an ICD-9 code for history of fracture, 12.3% of patients had osteoporosis, 5.4% of patients had nephrolithiasis/nephrocalcinosis, and 9.2% of patients had depression or anxiety.

**Table 2:** Baseline characteristics of patients undergoing parathyroidectomy due to primary hyperparathyroidism, 2002–2011.

	2002	2003	2004	2005	2006	2007	2008	2009	2010	2011	All	*P* value
N	13,333	12,550	13,203	11,604	10,740	9,163	11,449	8,549	9,057	9,934	109,583	
Age, median (IQR)	62 (52–72)	62 (52–71)	61 (52–72)	62 (53–72)	62 (52–72)	63 (53–72)	62 (53–71)	63 (53–72)	63 (53–72)	63 (54–72)	62 (52–72)	0.012
Age, %												
0–44	12.81	11.55	10.97	11.10	10.84	10.57	10.92	10.43	10.98	9.42	11.04	0.002
45–64	43.41	45.21	46.43	43.36	44.46	42.71	45.64	43.76	43.53	43.94	44.34	0.582
65-	43.78	43.24	42.60	45.55	44.70	46.73	43.44	45.81	45.49	46.64	44.62	0.052
Female, %	74.72	75.58	77.06	77.30	75.99	78.14	77.62	76.87	78.13	78.00	76.83	0.002
Race, %												
White	58.96	55.66	60.61	61.39	57.13	53.10	65.38	58.09	62.72	65.91	59.91	0.021
Black	9.40	10.20	12.08	8.13	8.30	10.48	8.14	9.59	15.10	12.23	10.27	0.673
Hispanic	2.98	5.14	3.40	4.82	5.96	5.87	4.60	6.67	7.46	6.73	5.17	0.002
Others	2.37	1.36	1.32	2.45	2.19	2.66	1.48	3.18	2.65	2.30	2.13	0.379
Missing	24.81	26.37	21.28	21.69	24.93	24.93	18.5	20.49	9.88	11.02	20.77	0.002
Region,%												
Northeast	23.21	21.81	25.28	23.24	18.91	17.91	33.02	21.30	27.75	27.65	24.09	0.378
Midwest	23.03	22.77	20.61	23.11	21.95	22.45	19.67	26.16	21.92	24.27	22.48	0.851
South	32.10	35.30	33.14	28.24	32.20	28.17	23.65	27.40	27.19	27.29	29.77	0.075
West	21.65	20.12	20.97	25.40	26.93	31.47	23.66	25.14	23.14	20.79	23.66	0.547
Hospital status, %												
Rural	6.83	6.38	4.59	5.39	4.81	6.75	6.03	6.45	8.49	3.03	5.83	0.742
Urban nonteaching	29.70	29.70	34.99	40.51	33.93	32.89	28.97	30.15	30.01	26.11	31.82	0.294
Urban teaching	63.47	63.92	60.43	54.10	61.26	60.36	65.00	63.40	61.50	70.86	62.35	0.297
Hospital bed size, %												
Small	10.14	10.76	8.58	7.22	8.02	6.56	5.41	8.72	7.35	7.62	8.14	0.142
Medium	21.38	22.62	17.61	20.48	21.46	26.05	19.14	23.11	20.09	16.83	20.75	0.655
Large	68.47	66.62	73.81	72.30	70.53	67.38	75.45	68.17	72.55	75.55	71.10	0.256
Payer, %												
Medicare	43.65	42.88	42.93	45.43	44.70	47.34	43.46	44.70	44.87	47.55	44.59	0.053
Private	49.21	49.86	49.46	46.77	46.06	43.27	48.51	41.78	40.97	42.43	46.30	<0.0001
Other	7.14	7.26	7.60	7.80	9.25	9.40	8.04	13.53	14.16	10.03	9.11	<0.0001
Comorbidities, %												
Diabetes	13.23	15.33	14.25	14.71	14.82	16.19	14.66	14.77	17.29	17.98	15.19	0.0001
Hypertension	43.29	47.53	49.82	50.71	48.90	54.55	51.42	52.64	56.22	54.56	50.51	<0.0001
Osteoporosis	9.14	9.84	10.75	14.11	12.45	13.34	15.12	15.10	12.56	12.07	12.25	<0.0001

Abbreviations: IQR, interquartile range.

**Table 3:** Most common single diagnosis codes of patients undergoing parathyroidectomy due to primary hyperparathyroidism, 2002–2011.

	ICD-9-CM codes	Description	%
1	252.0X	Hyperparathyroidism	82.26
2	227.1	Benign neoplasm of parathyroid gland	62.87
3	401.9	Unspecified essential hypertension	49.38
4	530.81	Esophageal reflux	15.20
5	275.42	Hypercalcemia	14.10
6	250.00	Diabetes mellitus without mention of complication, type II or unspecified type, not stated as uncontrolled	13.73
7	733.00	Osteoporosis, unspecified	11.72
8	272.4	Other and unspecified hyperlipidemia	11.04
9	272.0	Pure hypercholesterolemia	9.01
10	226	Benign neoplasm of thyroid glands	7.78
11	244.9	Unspecified acquired hypothyroidism	6.54
12	V15.82	Personal history of tobacco use	6.50
13	305.1	Tobacco use disorder	6.34
14	311	Depressive disorder, not elsewhere classified	5.61
15	241.1	Nontoxic multinodular goiter	5.56
16	278.00	Obesity, unspecified	5.37
17	493.90	Asthma, unspecified type, unspecified	5.34
18	414.01	Coronary atherosclerosis of native coronary artery	5.24
19	241.0	Nontoxic uninodular goiter	4.26
20	715.90	Osteoarthrosis, unspecified whether generalized or localized, site unspecified	4.12

The frequency was examined using single diagnosis code except “252.0 Hyperparathyroidsim”, whose code has divided to sub-codes during the study period.

### Parathyroidectomy Rates and Regional Variation

**Fig 2:**
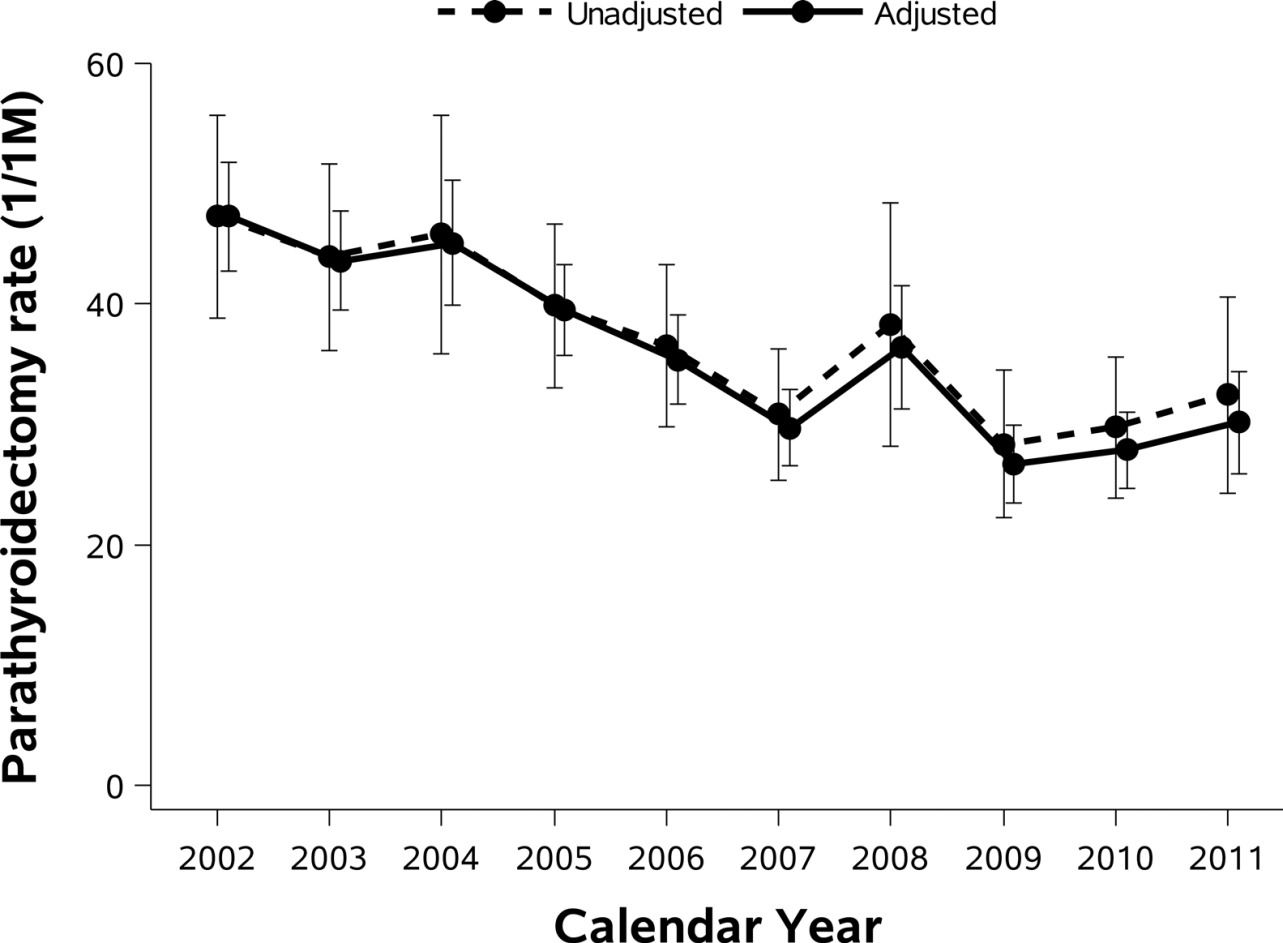
Unadjusted and adjusted inpatient parathyroidectomy rates with 95% CI for primary hyperparathyroidism. We calculated parathyroidectomy rates adjusted for age group and sex using the 2002 as standard population.

**Fig 3:**
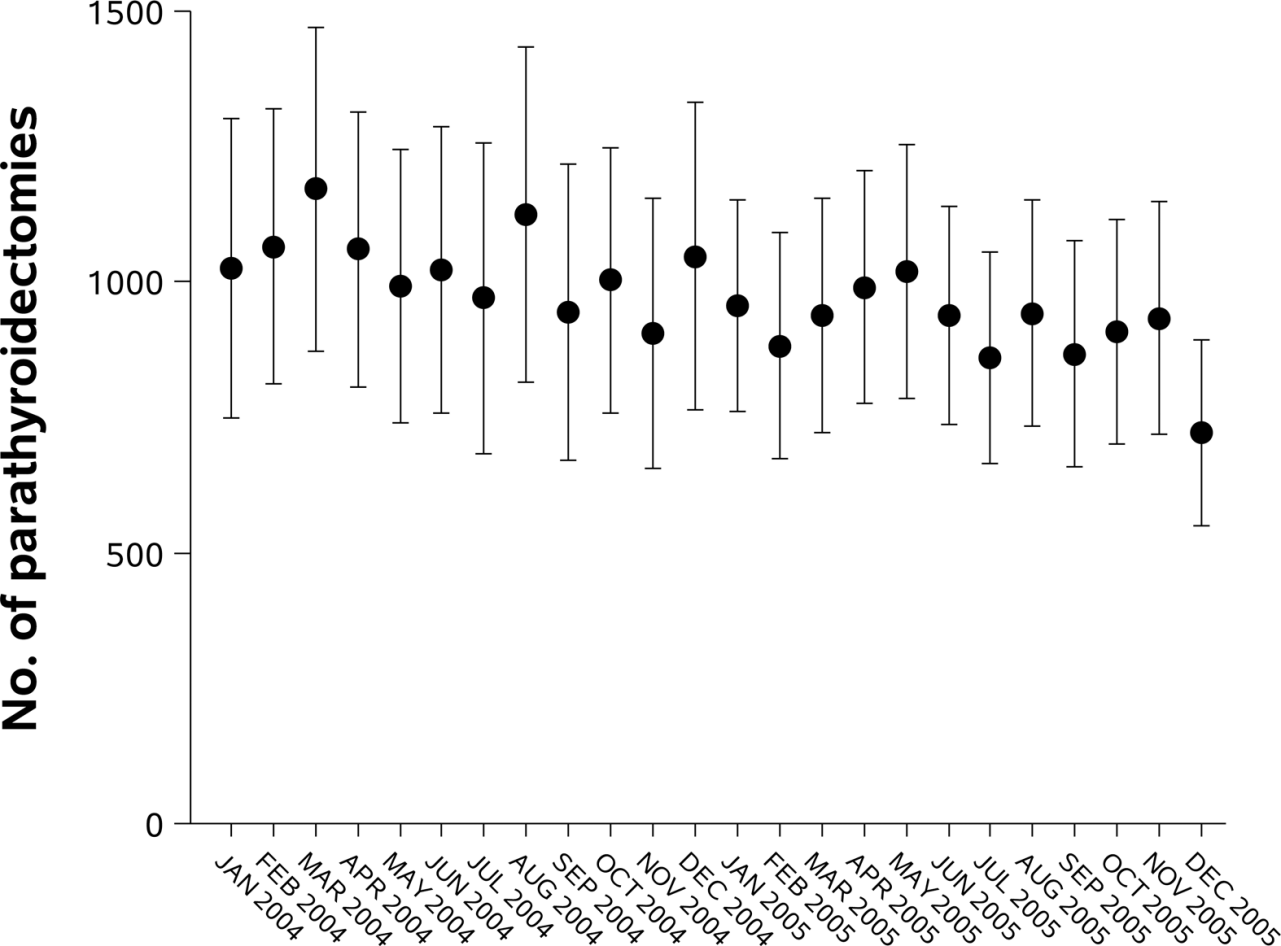
Estimated number and 95% CI of inpatient parathyroidectomies for primary hyperparathyroidism between 2004 and 2005 (described in monthly intervals).

**Fig 4:**
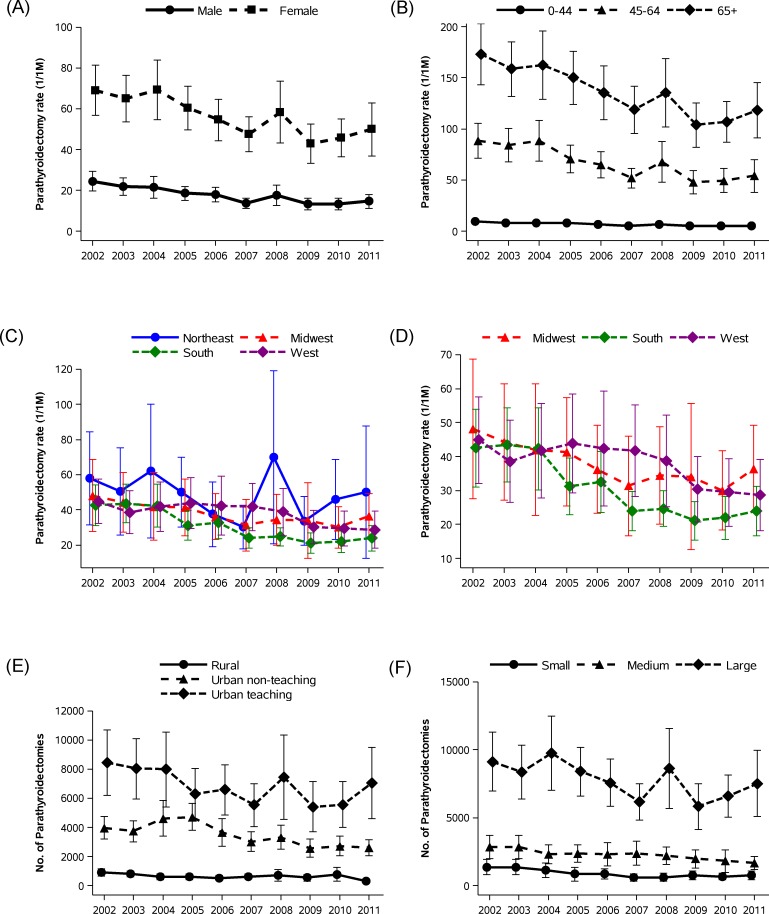
Inpatient parathyroidectomy rate and 95% CI for primary hyperparathyroidism by (A) sex, (B) age group, (C and D) region, and estimated number and 95% CI of parathyroidectomies by (E) hospital’s teaching status, and (F) hospital’s bed size.

Fig 2 shows unadjusted and adjusted parathyroidectomy rates in each calendar year. Parathyroidectomy rates declined between 2002 and 2011. The number of parathyroidectomies and adjusted parathyroidectomy rates decreased from 13,203 cases and 48.3 cases/million person-years in 2004 to 9163 cases and 31.7 cases/million person-years in 2007, and was sustained thereafter (in 2011: 9934 cases and 32.3 cases/million person-years). Dividing a portion of the study time frame in monthly intervals, we found that number of parathyroidectomies started to decrease from March 2004 and then continuously decreased over time (*P* for trend <0.0001, Fig 3). We determined unadjusted parathyroidectomy rates by age group and sex in each year (Fig 4). Although parathyroidectomy was more prevalent in women relative to men, similar trends over time were observed. Inpatient parathyroidectomy rates declined across the age groups, however a steeper decline was observed in older patients (>65 years; *P* for interaction <0.0001). We also calculated unadjusted parathyroidectomy rates by region. In the Northeast, there was a brief surge in parathyroidectomy rates with wide confidence intervals, especially in 2008. The 2008 rise was observed principally in hospitals classified as larger in size, urban and teaching (these features are not mutually exclusive). Although inpatient parathyroidectomy rates declined over time across the regions, a steeper decrease was observed in the South compared to other regions (*P* for interaction <0.0001). The regional difference remained after adjustment for age group and sex.

### In-hospital Mortality, Disposition, and Hospital Costs

Overall in-hospital mortality rates were 0.08%: 0.02% in patients younger than 65 years and 0.14% in patients 65 years and older, with no clear trend over time. Only 1.3% of patients developed postoperative hypocalcemia, with even fewer patients developing this complication in more recent years (*P* for trend = 0.0004). Post-operatively, almost 95% of patients were discharged home, while fewer than 3% were discharged to a skilled nursing facility or intermediate care. These proportions were stable over time. The median duration of the hospitalization for parathyroidectomy was 1 day (25^th^, 75^th^ percentile, 1 to 2 days; S1 Fig), while the median hospital costs steadily increased between 2002 and 2011 ($4,076 in 2002 *versus* $6,950 in 2011; *P* for trend <0.0001).

## Discussion

Using a representative inpatient national cohort in the US, we found that the rate of inpatient parathyroidectomy for pHPT has decreased in recent years. This declining trend was observed across age groups, both sexes, and all regions. The rate declined more steeply in the South compared to other regions, and comparably among age groups, men and women. Notably, in-hospital mortality rate after parathyroidectomy was below 0.1%, and postoperative hypocalcemia developed infrequently.

To our knowledge, this is the first report on inpatient parathyroidectomy for pHPT across the entire US. In contrast to the existing literature [1,13–17], we used the NIS to determine rates of parathyroidectomy across the population, allowing us to obtain rates and clinical characteristics of inpatient parathyroidectomy among a broader spectrum of patients regardless of age, region, or insurance type. Wu *et al*. reported a prevalence rate of parathyroidectomy for pHPT ranging from 3.3 per 100,000 for California to 5.8 per 100,000 for New York using data derived from private health insurers [22].

We observed that the inpatient parathyroidectomy rate declined between 2002 and 2011. Although a single report suggested that the incidence of pHPT might have declined in the early 1990s in the US [13], this finding has not been confirmed. In more recent data [16,17], the incidence of pHPT appeared to be increasing through the late 1990s and sustained, and the prevalence of pHPT has increased. These recent changes might be related to an increase in PTH testing and bone mineral density (BMD) measurement, particularly in postmenopausal women [17]. Reasons for declining rates of inpatient parathyroidectomy are likely multifactorial. The ability to perform the parathyroidectomy procedure for patients with pHPT as an outpatient is likely to explain a sizeable fraction of the decline in inpatient parathyroidectomy rates [23–24]. Stack *et al*. reported that parathyroid surgery was increasingly being performed in the outpatient setting between 2005 and 2010 using data from the University Health System Consortium (UHC) [23]. While the UHC consists of 112 academic medical centers and more than 250 of their affiliated hospitals and is broadly representative of nonprofit academic medical centers in the US, results from UHC cannot be extrapolated to all US hospitals or medical centers. Another plausible explanation for the decline in parathyroidectomy is the far more frequent testing for vitamin D deficiency. With the improved assay, faster results, and wider availability, routine testing of 25-hydroxy vitamin D has increased sharply over the past 10–15 years [25]. Availability of the 25-hydroxy vitamin D assay may have helped clinicians more easily distinguish patients with pHPT from patients with (nutritional) secondary HPT. Once recognizing vitamin D deficiency as a potential cause of hyperparathyroidism, fewer patients might have proceeded to imaging studies and ultimately to surgery. Trends in drug therapy may also have affected parathyroidectomy rates. Prescriptions for post-menopausal hormone therapy, which has been shown to reduce bone resorption, decrease serum calcium, and increase BMD in women with pHPT [26–28], decreased by >50% in 2002–2004 after the Women’s Health Initiative trials demonstrated that hormone therapy was not cardioprotective [29–30]. This sizeable, one-time coordinated withdrawal of hormone therapy may have “unmasked” more pHPT and worsened pre-existing pHPT. We wonder if a one-time peak in pHPT incidence could have contributed to the short-term higher rates of parathyroidectomy observed in the 2002–2005 time period. Oral bisphosphonates to treat osteoporosis became available in 1995 and prescription rates increased steadily before plateauing in 2006 [31,32]. Perhaps most importantly, the calcimimetic cinacalcet was approved for sHPT by the US FDA in March 2004. Although cinacalcet did not receive its approval for pHPT until February 2011, our findings from 2004–05 suggest that some patients with pHPT might have used cinacalcet (off-label) before the 2011 approval. Cinacalcet has been known to normalize serum calcium concentrations in patients with pHPT [33–36], usually with a dose of 30 mg twice daily [35]. To our knowledge, there are few data regarding improved bone density, or reduced incidence of fracture or kidney stones following treatment of pHPT with cinacalcet, however it could be one management option for patients who refuse parathyroidectomy or who are unable to undergo surgery due to cardiovascular or other comorbidities. Also, the 2008 International Workshop on the management of asymptomatic pHPT produced important modifications of two items in the guidelines: hypercalciuria was no longer regarded as an indication for parathyroidectomy and impaired kidney function was defined by creatinine clearance below 60 mL/min [11]. Considering that hypercalciuria was highly predictive of operative management in pHPT [15], adoption of these guidelines may have sustained lower parathyroidectomy rates through subsequent years.

We observed regional variation in the frequency of inpatient parathyroidectomy: a rise in 2008 in the Northeast region and more prominent decline from 2002–2011 in the South. We also found that a rise in 2008 was limited to larger, urban, teaching hospitals (Fig 4), only in Northeast region (S2 Fig). The NIS includes all hospitalization records from the “sampled” hospitals and provides the estimates from the “sample.” We assume that this abrupt rise might originate from a specific time and location and might be attributed to sampling effects from the NIS dataset. We were somewhat surprised to find the steeper declining trend in the South. In addition, the South *versus* other region trend is more prominent among the older age group, particularly between 2003–2007 (S3 Fig). One possible explanation could be varying regional penetrance of minimally invasive surgical techniques. Also, given the higher proportion of African Americans in southern states, and the high prevalence of obesity, metabolic syndrome and type 2 diabetes, more patients in southern states may have been identified as having vitamin D deficiency, with nutritional secondary HPT.

We found in-hospital mortality rates to be extremely low. The mortality rate was 0.02% in patients younger than 65 years and 0.14% in patients 65 years and older. These findings are in line with a previous report from Thomas *et al*. [37]: among patients who underwent parathyroidectomy for pHPT, in this study, 30-day mortality rate was significantly higher in elderly patients (≥80 years) than younger patients (0.8% *versus* <0.1%). Also, in-hospital mortality was far lower than that associated with parathyroidectomy for renal hyperparathyroidism [38]. Taken together, parathyroidectomy for pHPT is a safe procedure, though the risks are slightly higher for elderly patients, particularly those over 80 years old.

Although our study used a well-characterized database, we must acknowledge some important limitations. We had no laboratory or imaging data. Therefore, we could not determine any of the metrics of pHPT severity, including actual PTH values, the degree of hypercalcemia, hypophosphatemia or hypomagnesemia, bone density or the burden of nephrolithiasis. As such, we could not determine the reason or reasons for parathyroidectomy, and the rationale for whether the procedure should be performed as an inpatient, observation stay, or outpatient. We could not obtain patient-specific follow-up information to determine longer-term consequences of parathyroidectomy. As the NIS tracks hospital admissions and not patients *per se*, we may have included in our analysis some patients who underwent repeat parathyroidectomy, although these cases likely represent a very small minority of cases overall. Also, the NIS contains hospital discharge summary data from inpatients only. Therefore, operations that might have been performed on an outpatient basis would not be included in this study. We recently described parathyroidectomy rates for secondary, rather than primary hyperparathyroidism, and found non-declining rates, despite the availability of medications, including an oral calcimimetic indicated for use. We suspect that fewer patients with secondary hyperparathyroidism undergo outpatient parathyroidectomy, given 1) the comorbidity burden of patients with end-stage renal disease; and 2) the risk of symptomatic hypocalcemia associated with hungry bone syndrome.

In summary, using a nationally representative database, we examined the rates of inpatient parathyroidectomy for pHPT over the last decade. We found declining rates of parathyroidectomy over the last 10 years of available data. In-hospital mortality rates were very low, although mortality rates for elderly patients were relatively higher. With an increasing proportion of women and men living into their ninth and tenth decades, and the risks of hip fracture and other major complications that may develop in affected patients who are untreated, we may need to re-evaluate indications for parathyroidectomy in patients who are asymptomatic. As with nearly all surgical treatments, physicians and surgeons need to balance longer-term benefits on survival, complications and health status with the risks of perioperative death and other short-term complications.

## Supporting Information

S1 Fig The distribution of length of hospital stay in 2002–2011. (TIFF)

S2 Fig Estimated number and 95% CI of parathyroidectomies according to region. (A) among urban teaching hospitals and (B) among large bed-size hospitals.
(TIF)

S3 Fig Temporal trends in parathyroidectomy rate and 95% CI for primary hyperparathyroidism according to age by region. (A) Northeast, (B) Midwest, (C) South, and (D) West.
(TIF)

S1 Table Diagnosis and procedure codes used to define study population, outcomes, and covariates (DOCX)
