# Supplementary figures and images for: Declining Rates of Inpatient Parathyroidectomy for Primary Hyperparathyroidism in the US
Source: PLoS One. 2016 Aug 16;11(8):e0161192. doi: 10.1371/journal.pone.0161192 (PMC4986953; doi:10.1371/journal.pone.0161192)

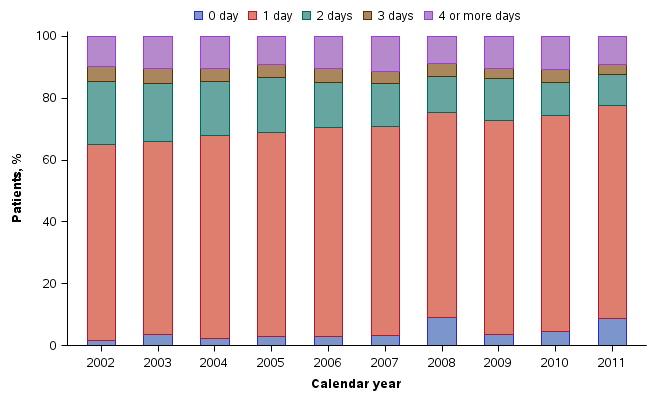

Supplement: S1 Fig — (TIFF) [file pone.0161192.s001.tiff]

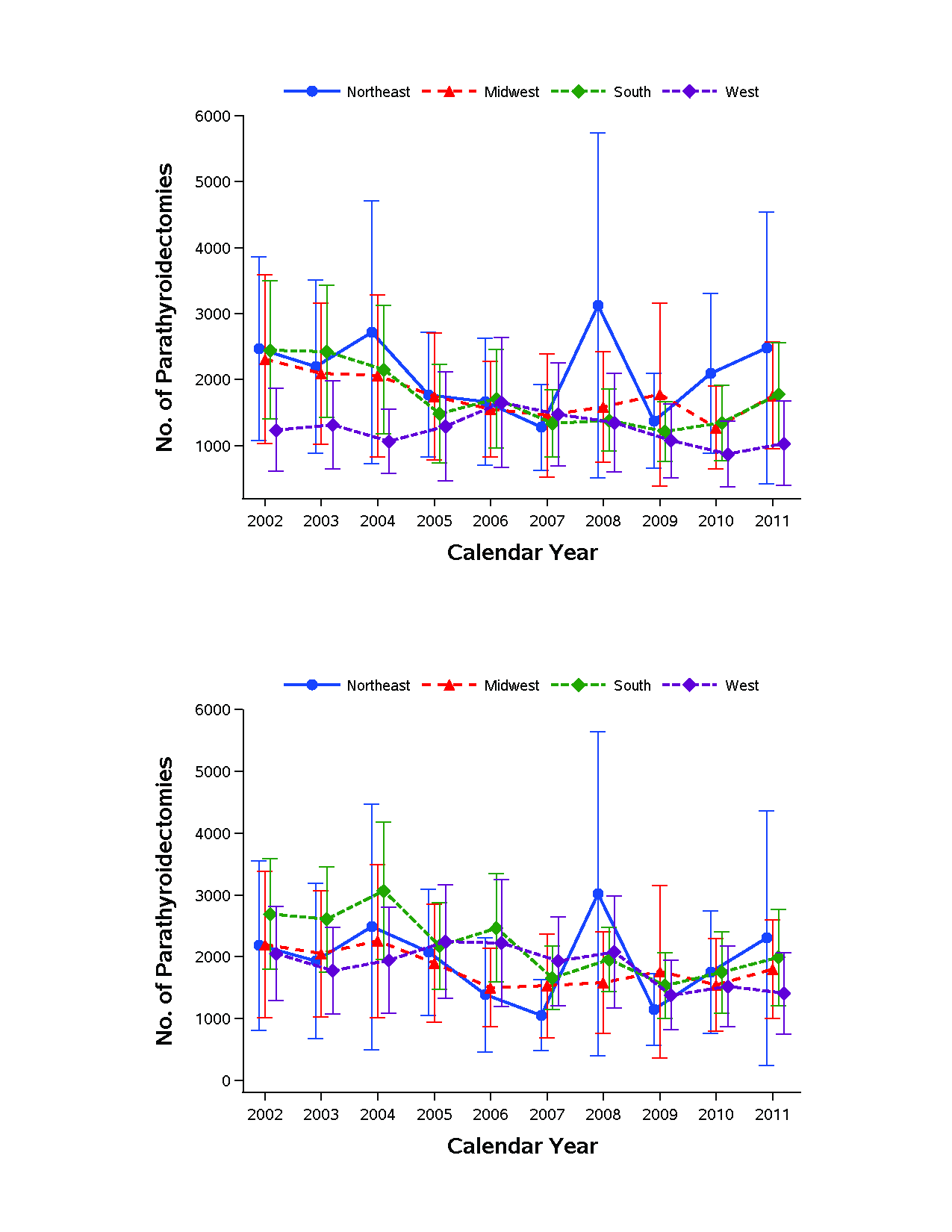

Supplement: S2 Fig — (A) among urban teaching hospitals and (B) among large bed-size hospitals. (TIF) [file pone.0161192.s002.tif]

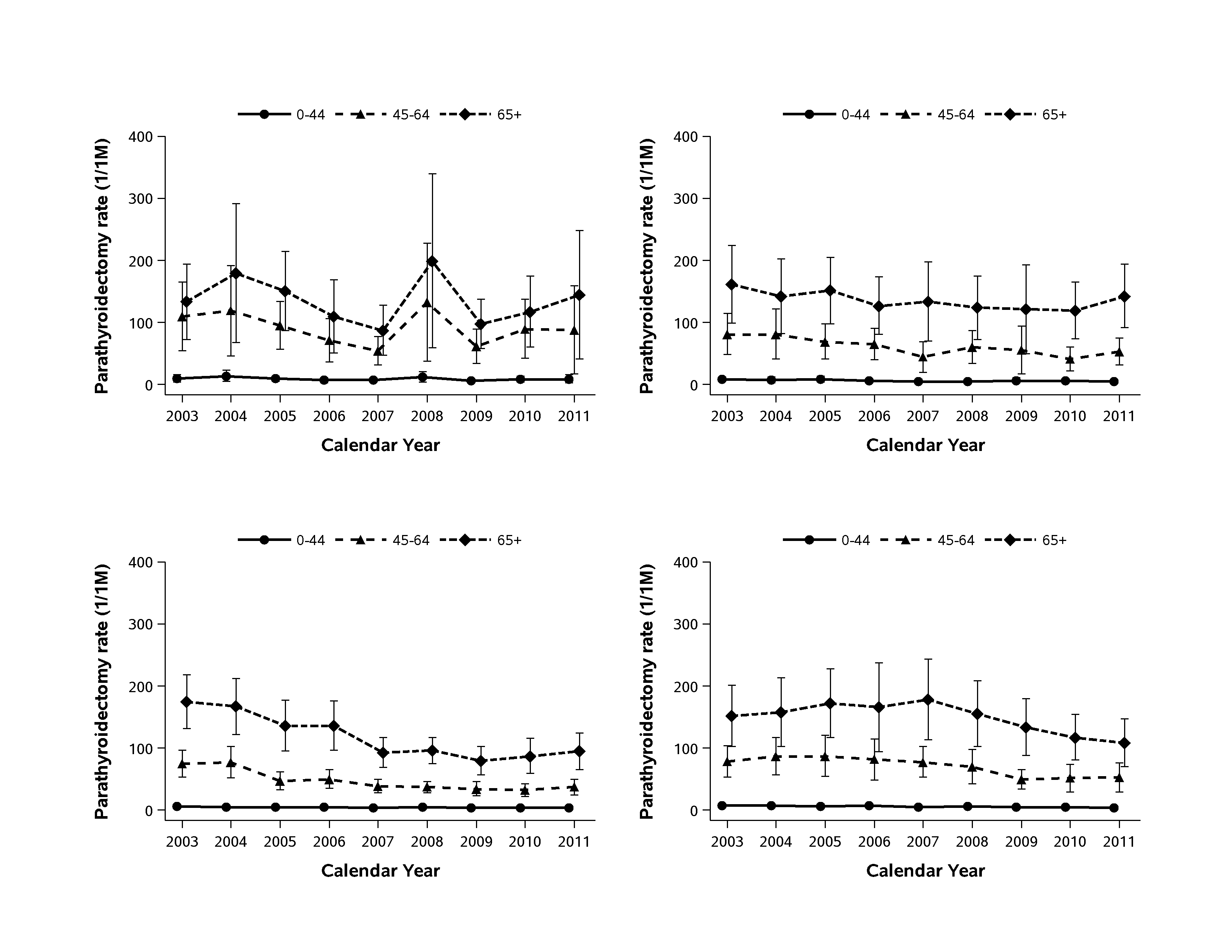

Supplement: S3 Fig — (A) Northeast, (B) Midwest, (C) South, and (D) West. (TIF) [file pone.0161192.s003.tif]
