# Supplementary material for: Declining Rates of Inpatient Parathyroidectomy for Primary Hyperparathyroidism in the US
Source: PLoS One. 2016 Aug 16;11(8):e0161192. doi: 10.1371/journal.pone.0161192 (PMC4986953; doi:10.1371/journal.pone.0161192)
Supplement: S1 Table — (DOCX) [file pone.0161192.s004.docx]

**S1 Table. Diagnosis and procedure codes used to define study population, outcomes, and covariates.**

| Description | ICD-9 CM codes |
| --- | --- |
| Parathyroidectomy | 06.81 06.89 |
| End-stage renal disease/  Renal failure  Dialysis/  Kidney transplantation | 403.01 403.11 403.90 403.91 404.02 404.03 404.12 404.13 404.92 404.93 585.3 585.4 585.5 585.6 585.9 586 996.81 V42.0 V45.11 V45.12 V56.X 39.95 54.98 |
| Secondary hyperparathyroidism | 588.81 |
| Thyroid cancer | 193 |
| Parathyroid cancer | 194.1 |
| Primary hyperparathyroidism | 252.00 252.01 252.08 |
| Multiple endocrine neoplasia | 258.0X |
| Hypercalcemia | 275.42 |
| Nephrolithiasis/Nephrocalcinosis | 592.X V13.01 |
| Other parathyroid disease | 252.02 252.1 252.8 252.9 |
| Parathyroid nodule | 227.1 |
| Diabetes mellitus | 249.X 250.X 648.0X 775.1 |
| Hypertension | 401.X 402.X 403.X 404.X 405.X 437.2 642.0X 642.1X 642.2X 642.7X 642.8X 642.9X |
| Osteoporosis | 733.0X |
| Fracture | 733.1X 733.93 733.94 733.95 733.96 733.97 733.98 733.99 80X 81X 82X 905.0 905.1 905.2 905.3 905.4 905.5 E88.7 V13.51 V13.52 V15.51 V54.1X V54.2X V66.4 |
| Depression | 300.4 301.12 309.00 309.1 311 |
| Anxiety | 293.84 300.00 300.02 300.09 309.24 309.28 |
